# Central Sugar Metabolism and the Cell Wall

**DOI:** 10.1128/mbio.02104-22

**Published:** 2022-09-12

**Authors:** Hajar Yaakoub, Jean-Paul Latgé, Nicolas Papon

**Affiliations:** a Université Angers-Université Brest Infections Respiratoires Fongiques, Structure Fédérative de Recherche “Interactions Cellulaires et Applications Thérapeutiques,” Angers, France

**Keywords:** *Aspergillus*, host-pathogen interaction, sugar metabolism, cell wall, virulence

## Abstract

The human opportunistic pathogen Aspergillus fumigatus is recognized for its versatile cell wall when it comes to remodeling its components in adaptation to external threats, and this remodeling renders it refractory to antifungals targeting cell wall biosynthesis. A specific role for general sugar metabolism in the regulation of the synthesis of cell wall polymers has been previously demonstrated. Delving deeper into central sugar metabolism may reveal unexpected fundamental aspects in cell wall construction, as shown by the work of Zhou and coworkers (Y. Zhou, K. Yan, Q. Qin, O.G. Raimi, et al., mBio 13:e01426-22, 2022, https://doi.org/10.1128/mbio.01426-22) on the roles of the phosphoglucose isomerase of A. fumigatus in cell wall biosynthesis.

## COMMENTARY

Two of the main issues that drive the severeness of infections caused by Aspergillus fumigatus are (i) being equipped with virulence traits and (ii) acquisition of resistance to the first-line drugs, triazoles. Research on the pathobiology and ecology of A. fumigatus has recognized its highly versatile nature as the leading factor driving its pathogenic behavior and saprophytism. This species, once termed “chameleon,” can adapt to whatever harnessing condition is taking place in a hostile environment (1, 2). This potential derives to a great extent from the plasticity of components of the A. fumigatus cell wall, which is mostly made up of polysaccharides (chitin, β-1,3-glucan, α-1,3-glucan, galactosaminogalactan, and galactomannan). In fact, A. fumigatus cell wall structures can dynamically reorganize according to growth demands and host threats (1) and have been recognized as drug targets for a long time since they are absent in mammals. The number of drugs targeting the fungal cell wall is still very limited. However, the slow emergence of azole-resistant strains over the last 2 decades may lead to increased use of cell wall drugs like echinocandins (3), which are noncompetitive drugs that inhibit β-1,3-glucan synthesis (4). While echinocandins remain one of the first-line drugs used against pathogenic yeasts, they only constitute the second-line defense against A. fumigatus. The reason for this is that the determination of an echinocandin *in vitro* efficacy is often questionable due to the large influence of medium composition, in addition to the manifestation of a “paradoxical effect” that entails a reduction in caspofungin efficacy at concentrations higher than the MIC (5, 6). In this regard, one could envision that sugar content in the external medium also impacts drug efficacy, simply because the metabolic sugar effect could control the central biochemical pathways that provide the end products for cell wall polysaccharides.

**FIG 1:**
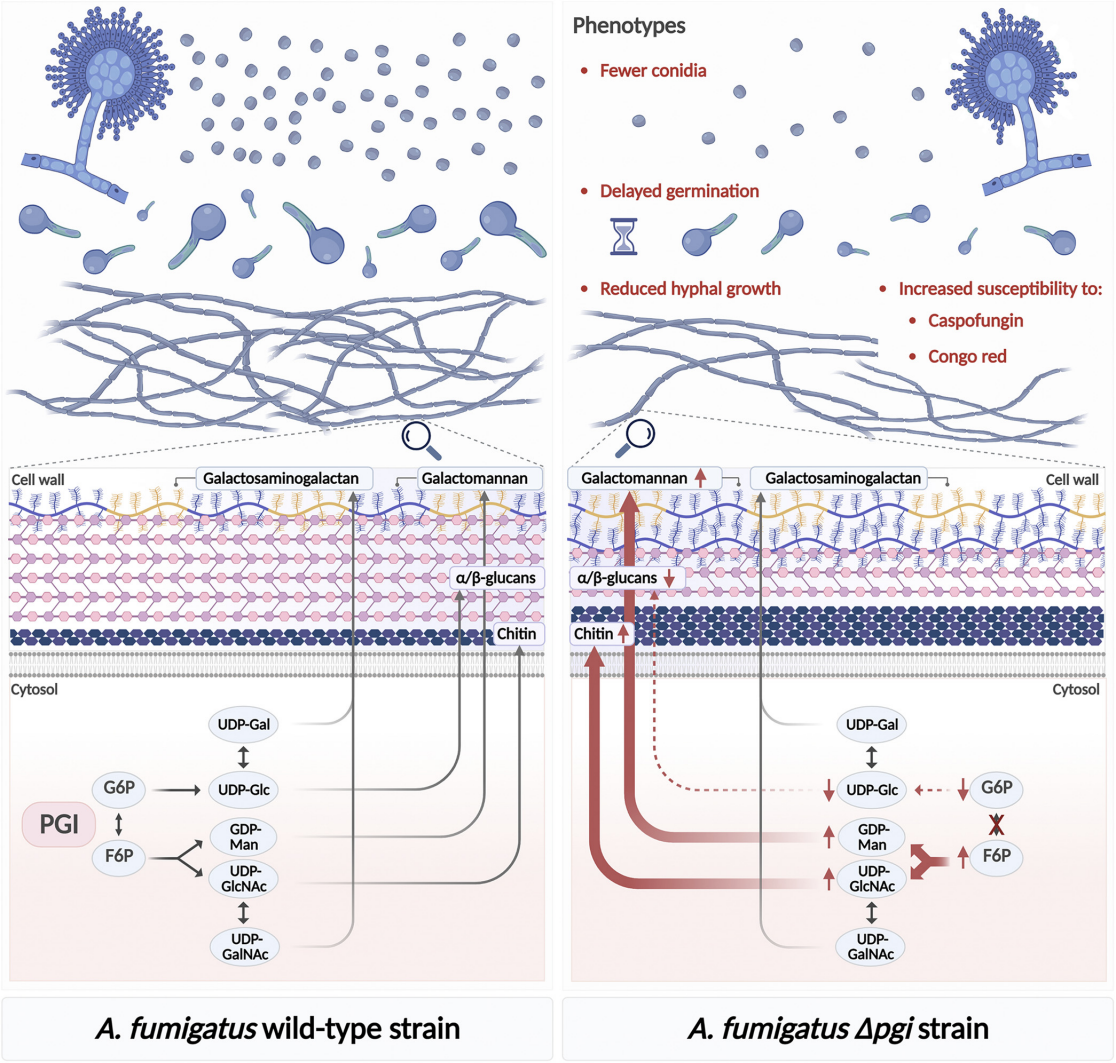
Involvement of PGI in sugar metabolism, cell wall biosynthesis, and morphogenesis in A. fumigatus. In the wild-type strain of A. fumigatus, PGI catalyzes the interconversion of glucose-6-phosphate (G6P) to fructose-6-phosphate (F6P). This step feeds into the production of sugar nucleotides like UDP-glucose (UDP-Glc), UDP-galactose (UDP-Gal), GDP-mannose (GDP-Man), UDP-*N*-acetylglucosamine (UDP-GlcNAc), and *N*-acetylgalactosamine (UDP-GalNAc), which serve as building blocks for cell wall components such as glucans, chitin, galactomannan, and galactosaminogalactan. When devoid of PGI (Δ*pgi*), the fungus exhibits an imbalance in sugar intermediates, including reduced G6P and increased F6P, which likewise impacts the production of relative sugar nucleotides and the derived cell wall components. The increased susceptibility of the Δ*pgi* mutant to caspofungin and Congo red also reflects impairment in the cell wall. The Δ*pgi* mutant showed disrupted morphogenesis, which is probably in relation to the role of PGI in sugar metabolism and cell wall biogenesis.

Glucose-6-phosphate isomerase, also designated phosphoglucose isomerase (PGI; EC 5.3.1.19), is one of the cardinal enzymes in sugar metabolism. PGI is an aldose-ketose isomerase that catalyzes the interconversion of glucose-6-phosphate and fructose-6-phosphate, a step that feeds into vital pathways including glycolysis, gluconeogenesis, and sugar nucleotide biosynthesis (7) (Fig. 1). This obviously explains the occurrence of PGI across all domains of life. Consistently, the primary role of PGI in glucose metabolism is universally conserved. The conservation also extends to catalytic and binding sites, as substantiated by crystal structures determined for PGI from the eukaryotic and prokaryotic worlds. Besides their primary roles in glucose metabolism, PGI displays moonlighting functions. In fungi, research on the moonlighting functions of PGI has shown that they are morphogenesis centered, although species-specific functions still exist. For instance, PGI is responsible for sporulation in Saccharomyces cerevisiae (8) and the formation of fruiting bodies in Coprinus macrorhizus (9). In Cryptococcus neoformans, the enzyme contributes to the production of virulence factors (i.e., capsule and melanin) and tolerance to stress, including cell wall stress (10). In Fusarium graminearum, PGI is indispensable for hyphal growth, germination, septal formation, phytopathogenicity, and resistance to osmotic stress (11). Also, hyphal polarity and conidiation in Aspergillus nidulans are positively regulated by PGI (12). As for Aspergillus flavus, while PGI is not required for hyphal development, it affects germination, conidiation, sclerotia formation, virulence, and resistance to stress, including cell wall-disrupting agents (13). How PGI regulates fungal morphogenesis and how it affects the cell wall remain very fuzzy. In order to address these questions, Zhou et al. (14) used for the first time the human pathogenic filamentous fungus A. fumigatus.

In their manuscript, the authors characterized the *pgi* null mutant (Δ*pgi*) in A. fumigatus and embraced two intertwined functions of the A. fumigatus PGI (*Af*PGI) in (i) sugar metabolism and (ii) cell wall biosynthesis, which were compelling enough to explore the role of PGI in virulence and to solve its crystal structure (14) (Fig. 1). The first function was supported by the fact that deletion of *pgi* was lethal unless different carbon sources were present, such as both PGI substrates and products, glucose (0.1%) and fructose (1%). Second, and more importantly, the study provided evidence on fungal PGI-mediated regulation of precursors of cell wall components, such as UPD-glucose, UDP-*N*-acetylglucosamine, and GDP-mannose (precursors of glucan, chitin, and mannan, respectively). In fact, the Δ*pgi* mutant had reduced intracellular UDP-glucose (by 70%) but highly increased UDP-*N*-acetylglucosamine and GDP-mannose (by 185% and 225%, respectively), which consistently translated into decreased glucan contents and increased chitin and glycoproteins (from which mannan release was increased as well) (Fig. 1). As such, it was unsurprising that the deletion of *Afpgi* engendered higher sensitivity to caspofungin and Congo red (14). Similar findings were recently reported in Lentinula edodes, in which silencing of *pgi* entailed decreased expression of genes involved in β-1,3-glucan synthesis, whereas genes related to chitin synthesis were upregulated, all along with increased sensitivity to cell wall-disrupting agents (15). Salvage pathways to compensate for the halt of glucan production by upregulation of the other components have been repeatedly shown in the past (1). The high-osmolarity glycerol pathway and the cell wall integrity pathway constitute potential candidates, since both pathways are specifically rewired together toward cell wall-related functions in A. fumigatus, including biosynthesis and remodeling (16). Identification of the salvage pathway compensating for the lack of *Af*PGI and thereby its inhibitors may help circumvent the drawback of echinocandins, perhaps using combinatorial approaches. It would also be relevant to decipher the relationship between PGI and the fuelers of glucose-6-phosphate (i.e., the trehalose pathway and hexokinase).

With regard to the deteriorated conidiation, germination, and hyphal growth exhibited by the Δ*pgi* mutant, the study by Zhou and coworkers (14) also revealed the concept that sugar metabolism and cell wall biosynthesis functions are tightened together during growth and morphogenesis (Fig. 1). Being inspired by models in higher eukaryotes like plants and humans, where roles of UDP-glucose and UDP-*N*-acetylglucosamine in cellular signaling are pronounced (17–19), a hypothesis can be stated here that UDPs act as signaling molecules for fungal development, and so the imbalance in the UDP pool in the Δ*pgi* mutant resulted in defective growth. It should be emphasized that the impact of sugar nucleotides on fungal growth has never been tested in fungi, and so conducting related research becomes an afterthought. Another hypothesis also could not be disregarded, that the defective growth features in the mutant are secondary effects of impaired *de novo* cell wall biosynthesis caused by a compromised UDP-glucose pool. All these results led Zhang et al. to suggest that inhibition of PGI could constitute an effective therapy that culminates in abolishing sugar metabolism, cell wall biosynthesis, and development.

Based on the conclusive evidence supporting such targeting, Zhang et al. proceeded to solve the crystal structure of PGI so that it could be used to model docking of potential drugs. Interestingly, two structural differences were pointed out in *Af*PGI. First, the enzyme is unlike its counterparts in that it interacts with the substrate phosphate group using its neutral side chains, and second, it holds near the active site an alanine residue at position 211 that is conserved within pathogenic fungi but replaced with glutamine in the human ortholog. The first difference infers that *Af*PGI could be inhibited using pseudosubstrates harboring a neutral phosphate instead of the classical polar phosphate, which would improve membrane permeability. As for the second difference, the authors stated that alanine in *Af*PGI is small enough to leave a gap for a ligand between the residue and the active site, which may guarantee specificity for fungal inhibitors. Nonetheless, the druggability of PGI as a target remains uncertain until an appropriate inhibitor has been identified. Several fungal glycoenzymes, of which human counterparts exhibit similar activity, have been proposed as putative drug targets based on specifications in catalytic sites (20–22). Results probing their druggability, however, have not been obtained. Exploring such findings related to central sugar metabolism in molecular docking studies is an urgent but unmet need.

Overall, the study by Zhou et al. (14) has deciphered the complexity of the links between cell wall synthesis and central sugar pathways in fungi. On the applied level, their report propels additional challenges to the discovery of therapeutic targets related to biosynthetic enzymes involved in A. fumigatus cell wall construction. Their work, along with previous studies focused on PGI in other Aspergillus species (i.e., A. flavus and A. nidulans) (12, 13), also points out the magnitude to which ortholog enzymes may participate in differential regulation within taxonomically related molds, probably through unidentified signaling pathways where sugar nucleotide intermediates constitute the cues. The Zhang et al. study also underscores the adaptability of all fungi toward stress and growth inhibition, through which the production of chitin and β-glucan is increased and reduced, respectively. This gives rise to the question of what signals are responsible for such behavior. Is the increase in chitin production and loss of β-glucan needed for the fungus to become dormant or resistant to adverse conditions? This revives an interest in unveiling the mechanisms underlying this versatility and the importance of searching for fungal chitin synthesis inhibitors, many of which have been identified for chitin synthesis in insects (23).
